# Trends in Cardiovascular Diseases and Costs Among Type 2 Diabetes Mellitus (T2DM) Patients in Malaysia: A Cohort Study of 240,611 Public Hospital Inpatients

**DOI:** 10.7759/cureus.75531

**Published:** 2024-12-11

**Authors:** Noor Adilah Kamarudin, Sharifa Ezat Wan Puteh, Mohd Rizal Abd Manaf, Mohd Ridzwan Shahari

**Affiliations:** 1 Public Health, Hospital Canselor Tuanku Mukhriz, Kuala Lumpur, MYS; 2 Public Health, Ministry of Health Malaysia, Kuala Lumpur, MYS

**Keywords:** cardiovascular disease event, casemix, diagnosis related group, hospital admission, treatment cost, type 2 diabetes mellitus

## Abstract

Background: Identifying trends of hospital admissions and costs for cardiovascular disease events (CVDEs) is crucial for public health intervention and the economic burden for future clinical improvements and better outcomes. This study aims to define the admission trends and cost of CVDE among type 2 diabetes mellitus (T2DM) patients in Malaysia between 2014 and 2020.

Methodology: An ecological study was conducted using hospital admission data taken from the Casemix database in public hospitals in Malaysia. Hospital admission data for CVDE among T2DM patients were extracted for the period between 2014 and 2020. The cost data were retrieved from the Malaysian Disease Related Group (MalaysianDRG) costing section, and the median and total costs were calculated per CVDE per year. Descriptive statistical analysis and multiple logistic regression models were used to analyze trends and factors associated.

Results: A total of 240,611 T2DM admissions, representing 35.1% of 684,809 CVDE admissions, were included in this study. Among these, 32.9% were treated for myocardial infarction (MI), 20.1% for cerebrovascular accident (CVA), 19.4% for heart failure, 12.8% for ischemic heart disease (IHD), 8.2% for hypertensive heart disease (HHD), 5.6% for cardiomyopathy, and 1.0% for atherosclerosis and peripheral vascular disease (PVD). CVDE admissions were prevalent among males (59.2%) and associated with higher cost of admission (β = 1.13, *P* < 0.001), patients aged 40-49 years old had 24% high odd for high cost (β = 1.24, *P* < 0.001) compared to those aged 19-29 years. Compared to Malay, Chinese and other ethnicities were significantly associated with high cost (β = 1.13, *P* < 0.001). Patients with severity level III were 10 times more likely to have higher costs as compared to severity level I (β = 10.39, *P* < 0.001), 72.6% were admitted in less than five days, and 23.1% were less likely to incur high cost as compared to patients admitted more than five days (β = 0.769, *P* < 0.001). The trend of admissions is increasing each year, with the median total hospital expenditure higher in IHD patients with T2DM, which increased by 55.5% from 2014 to 2020 (from RM 4,187.98 to RM 6,510.43). This was followed by MI, which saw an 8% increase (from RM 3,881.80 to RM 4,211.18).

Conclusions: The findings of this research indicated cardiovascular disease (CVD) admission trends and costs increased substantially over the years and higher costs in dual noncommunicable diseases (NCDs). These findings underscore the urgent need for enhanced preventive strategies targeting high-risk populations, such as males, individuals with severe disease levels, and specific ethnic groups. Policies should emphasize lifestyle modification programs, early diagnosis of cardiovascular risks among T2DM patients, and cost-effective treatments to mitigate the growing financial burden. Furthermore, resource allocation must be adjusted to address the increasing demand for care, particularly for conditions like IHD and MI, ensuring equitable access to quality care while containing healthcare costs.

## Introduction

Diabetes is a serious chronic condition with major medical, social, and economic impacts, estimated to have affected. It is a metabolic disease characterized by insulin resistance and defective insulin secretion, leading to hyperglycemia [1]. According to the International Diabetes Federation (IDF), in 2021, approximately 10.5% (537 million) of adults aged 20-79 years worldwide had diabetes. This number is projected to rise to 12.2% (783 million) by 2045 if effective prevention measures are not implemented [2]. Type 2 diabetes mellitus (T2DM) affects 14.4% (95% confidence interval [CI] 12.51%-16.38%) of the Malaysian population [3]. The prevalence of diabetes in adults aged >18 years has increased from 11.2% in 2011 to 18.3% in 2019 [4]. This brings the total number of adults living with diabetes in Malaysia to 3.9 million. By 2025, the prevalence of Malaysian with T2DM is projected to be 7 million [3].

Cardiovascular disease (CVD) is estimated to affect 32.2% of the diabetic population globally [5]. It is a significant global health concern worldwide, particularly among individuals with T2DM [6]. Malaysia, like many other countries, has witnessed a surge in the prevalence of both T2DM and CVD over the past few decades [7]. The rising prevalence of T2DM in the country has heightened the need to understand the trend in hospital admission due to CVD among this population [8,9]. The intricate interplay between these two conditions amplifies the risk of hospitalization, imposing substantial burdens on healthcare systems. The American Diabetes Association (ADA) estimated in 2017 that T2DM incurred $237 billion in direct medical costs in the United States, with $37.3 billion of that amount attributed to cardiovascular-related expenses [1]. Mehta et al. [10] found that patients with T2DM and selected CVDs (stroke, transient ischemic attack [TIA], myocardial infarction [MI], unstable angina, and coronary revascularization) had significantly greater healthcare resource utilization and higher costs than patients with T2DM alone. King et al. [1] also observed significantly higher healthcare costs for patients with T2DM and atherosclerotic CVD (ASCVD) compared to patients with T2DM alone.

Data from the National Diabetes Registry Report 2013-2019 showed that the overall trend of comorbidities, including hypertension and dyslipidemia, increased among T2DM patients in Malaysia over the years. Additionally, the prevalence of CVD complications such as IHD, nephropathy, and retinopathy was also reported to be substantial [8,11]. Understanding the temporal pattern of hospital admission is crucial for several reasons. First, it provides valuable insights into the dynamic relationship between T2DM and cardiovascular complications over a specific period. Second, it offers an opportunity to evaluate the effectiveness of existing healthcare strategies and interventions aimed at reducing hospitalizations associated with coexisting comorbidities. This ecologic study aims to scrutinize the hospital admission trends attributable to CVD among individuals diagnosed with T2DM in Malaysia. The period between 2014 and 2020 is of particular interest, as it encompasses a critical timeframe marked by evolving healthcare policies before the pandemic, lifestyle changes, and advancements in medical interventions.

The admission trends and costs associated with CVD events among T2DM patients in Malaysia reveal significant healthcare challenges. A study indicated that approximately 60.4% of patients admitted for CVD events had T2DM, with acute MI and IHD being the most prevalent conditions [12]. The total treatment cost for CVD events was 4.8 million Ringgit Malaysia (RM) for diabetic patients, highlighting the economic burden of these conditions [12]. Additionally, a retrospective analysis showed that 20% of diabetic patients required hospitalization for coronary heart disease, emphasizing the critical link between diabetes and cardiovascular complications. Furthermore, the National Health and Morbidity Survey indicated that a significant proportion of the population has at least one cardiovascular risk factor, underscoring the need for targeted interventions. Overall, these findings illustrate the increasing trend of CVD admissions among T2DM patients, coupled with substantial healthcare costs, necessitating improved management strategies.

Given the scarcity of ecologic studies exploring the specific nexus in the Malaysian context, our research seeks to bridge this knowledge gap. By analyzing nationwide data on public hospital admissions, we aspire to contribute evidence-based perspectives that can inform policymakers, clinicians, and researchers alike. The outcome of this study may serve as a foundation for tailored interventions, resource allocation, and preventive measures aimed at mitigating the impact of CVDs among individuals living with T2DM. In this paper, we present a comprehensive examination of the trends and patterns of hospital admissions and costs due to CVD among T2DM patients in Malaysia from 2014 to 2020.

The novelty of this study lies in its comprehensive approach to analyzing nationwide hospital admission data within the Malaysian context, an area with limited ecologic studies exploring the specific intersection of T2DM and CVD. By leveraging a robust dataset, this study addresses a critical knowledge gap, providing evidence-based insights into the temporal trends, demographic factors, and economic burden of CVD in T2DM patients in Malaysia.

The benefits to public health are substantial. This study not only highlights the rising healthcare costs associated with T2DM and CVD but also underscores the need for tailored interventions targeting high-risk populations. Findings can inform policymakers on resource allocation, development of preventive strategies, and implementation of cost-effective management programs. Such interventions can help curb the rising prevalence of these dual noncommunicable diseases (NCDs), reduce hospitalizations, and alleviate the financial strain on Malaysia’s healthcare system. Ultimately, this study aims to serve as a foundation for enhancing public health outcomes and improving equity in healthcare delivery for individuals living with T2DM and CVD.

## Materials and methods

The researchers conducted an ecological study using data from the Casemix Unit, Ministry of Health, Malaysia. The data utilized in this study are confidential and are not publicly accessible, as they are owned and maintained by the Malaysian government. Access to these data requires formal permission from the relevant government authorities. The Casemix database provides details information on hospital admission associated with a wide range of health conditions in public hospitals in Malaysia. Hospital admission data were collected for the period between January 2014 and December 2020. The Casemix database contains hospital admission data for all types of diseases, including CVD, T2DM, and its complications. The age, sex, and diagnosis of patients were included in the database. We identified CVD admission using the tenth version of the International Statistical Classification of Diseases (ICD-10) system. All diagnostic codes for diseases of CVD (I10-I73) and T2DM (E12-E14) were used to identify all hospital admissions related to various types of CVD in public hospitals in Malaysia. The CVD patient dataset was checked using VLOOKUP with T2DM data to determine the prevalence of T2DM among CVD admissions. The CVDs considered in this study were classified based on the World Health Organization (WHO) classification of the circulatory system (I00-I99) in Chapter IX of ICD-10. The chapter contained the following blocks based on the category:

I00-I02 Acute rheumatic fever

I05-I09 Chronic rheumatic heart disease

I10-I15 Hypertensive heart disease*

I20-I25 Ischemic heart disease* (including acute myocardial infarction)

I26-I28 Pulmonary heart disease and disease of pulmonary circulation

I30-I52 Other forms of heart disease* (heart failure)

I60-I69 Cerebrovascular disease* (stroke)

I70-I79 Disease of arteries, arterioles, and capillaries* (arteriosclerosis)

I80-I89 Disease of veins, lymphatic vessels, and lymph nodes not elsewhere classified

I95-I99 Other and unspecified disorders of the circulatory system

The asterisk category is the CVD that is described in this study. Data for hospital admissions have been available from 2014 onward. Available data include patient demographics, clinical diagnoses, procedures, and duration of stay. The Casemix data are audited regularly to ensure their validity and accuracy. In addition to these routine audits, the dataset underwent further validation checks to confirm data consistency and reliability, including cross-referencing with hospital records and conducting exploratory analyses to identify and address potential anomalies or discrepancies before further analysis for this study.

Inclusion criteria

The following criteria were used to select patients for this study:

(1) All patients admitted to a public hospital with ICD-10 code related to CVDE (I10.0-I79.0) and T2DM and its complications (E11-E14.0) from January 1, 2014, to December 31, 2020

(2) Individual age more or equal to 18 years old

Exclusion criteria

The following criteria were used to exclude patients from the study:

(1) Patients who had advanced systemic cancer, COVID-19, Hepatitis B or C, HIV, and known major mental illness listed as the main diagnosis of their admission

(2) Admission for acute rheumatic fever and chronic rheumatic heart disease (I00-I09) and disease of veins, lymphatic vessels, and lymph nodes not elsewhere classified and other and unspecified disorders of the circulatory system (I80-I99.0)

This study excluded all hospital admissions with ICD-10 codes I00-I09 and I80-I99, which represent acute events and unspecific CVD to reduce the risk of misclassification bias in identifying the cause of hospital admissions.

Statistical analysis

Descriptive analysis was performed to reveal the composition of CVD between sex and different age groups. A multiple logistic regression model was used to determine the factors influencing the cost of treatment, utilizing the Malaysian DRG costing data. The analysis focused on factors that influence treatment costs, such as hospital type, sex, age, ethnicity, length of stay, severity level, and type of CVD events. All analyses were performed in SPSS version 26.0 (IBM Corp., Armonk, NY). A *P*-value < 0.01 was used to indicate statistical significance.

## Results

The total annual number of CVD cases in T2DM patients increased by 157.4%, from 25,715 in 2014 to 40,480 in 2020. This represents an increase in the hospital admission rate for all CVD patients of 185.2%, from 71,836 in 2014 to 133,008 in 2020. During the study period, the most prevalent CVDs were myocardial infarction (MI), followed by cerebrovascular accident (CVA, commonly known as stroke), heart failure (HF), ischemic heart disease (IHD), hypertensive heart disease (HHD), cardiomyopathy, and atherosclerosis, which accounted for 32.9%, 20.1%, 19.4%, 12.8%, 8.2%, 5.6%, and 1.0%, respectively (Table 1).

**Table 1:** Percentage of CVD among T2DM hospital admissions from the total number of CVD admissions per ICD-10 code during the study period. CVD, cardiovascular disease; T2DM, type 2 diabetes mellitus; ICD-10, International Statistical Classification of Diseases System Version 10.0; MI, myocardial infarction; NSTEMI, non-ST-elevation myocardial infarction; ACS, acute coronary syndrome; AF, atrial fibrillation; SVT, supraventricular tachycardia; CCF, congestive cardiac failure; SDH, subdural hemorrhage; CVA, cerebrovascular accident; MCA, middle cerebral artery; PVD, peripheral vascular disease

ICD-10 Code	Description	Percentage from the total number of CVDE admissions (%)
I10-I12.9	Hypertensive heart disease (controlled, uncontrolled, young hypertension, and hypertension with complications)	8.2
I20-I22.9	Myocardial infarction (acute MI, chronic MI, inferior MI, anterior MI, unstable MI, and NSTEMI)	32.9
I24-I25.9	Ischemic heart disease (acute coronary syndrome, two-vessel disease, three-vessel disease, and ACS with complications)	12.8
I40-I49.9	Cardiomyopathy and conduct disorder (dilated cardiomyopathy, atrial fibrillation [AF], atrial flutter, recurrent supraventricular tachycardia [SVT], atrial-venous block, and AF with complication)	5.6
I50-I52.9	Heart failure (acute heart failure, heart failure with complication, congestive cardiac failure [CCF], decompensated CCF, stable CCF, acute pulmonary edema, and ventricular failure)	19.4
I60-I69.9	Cerebrovascular accident (cerebral stroke, cerebellar infarct, subdural hemorrhage [SDH], acute SDH, chronic SDH, acute CVA with complication, hemiparesis, MCA infarct, and MCA bleeding)	20.1
I70-I73.9	Atherosclerosis and peripheral vascular disease (atherosclerosis, vascular disease acute limb ischemia, chronic limb ischemia, peripheral vascular disease [PVD], and PVD with complications)	1

**Figure 1:**
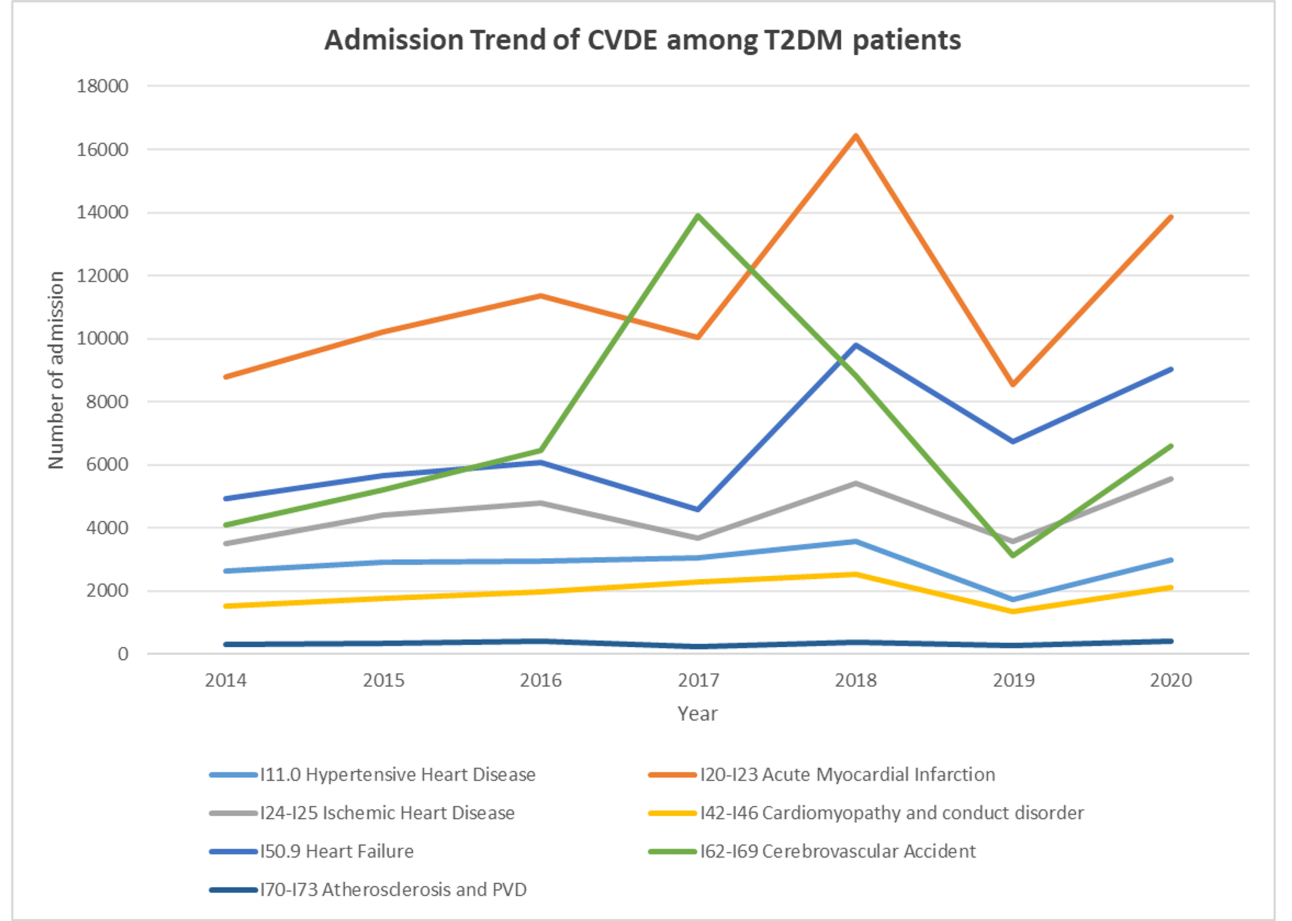
Hospital admission rates due to cardiovascular disease among type 2 diabetes mellitus patients between 2014 and 2020. CVDE, cardiovascular disease event; T2DM, type 2 diabetes mellitus; IHD, ischemic heart disease; MI, myocardial infarction; PVD, peripheral vascular disease

**Figure 2:**
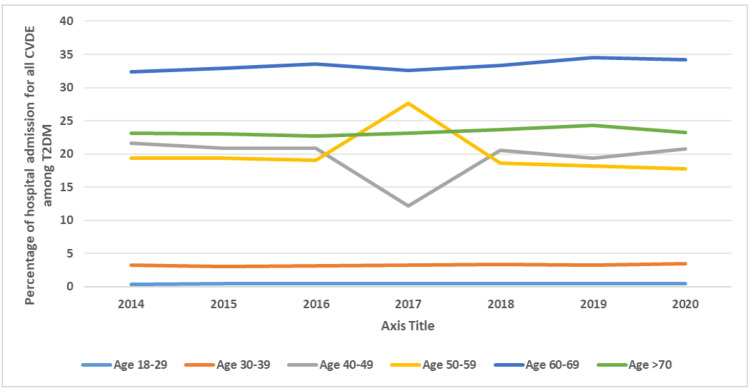
Percentage of hospital admissions for all CVDs among T2DM patients in Malaysia, stratified by age group. CVD, cardiovascular disease; CVDE, cardiovascular disease event; T2DM, type 2 diabetes mellitus

During the past seven years, the largest increase in the rate of hospitalization for CVD among T2DM patients was noted in HF with complications (I50-I52.9), followed by CVA (I60-I69.9), ischemic heart disease (I24-I25.9), and myocardial infarction (I20-I22.9), which increased by 1.8-fold, 1.6-fold, and 1.5-fold, respectively. Furthermore, the rate of hospitalization due to cardiomyopathy, atherosclerosis, and HHD increased by 140.1%, 131.6%, and 112.2%, respectively (Figure 1). Regarding the distribution of CVDE among different age groups, the 60-69 years age group accounted for 33.4% of the total number of CVDE cases during the study period, followed by the 70 years and above group with 23.4%, the 50-59 years group with 20.1%, and, lastly, the 40-49 years group with 19.5%. The CVDE rate among young patients showed an increase, with the 18-29 years age group rising by 1.7-fold from 2014 to 2020, followed by the 30-39 years age group, which increased by 1.1-fold. The increasing trend in patients aged 50-59 and the decreasing trend in patients aged 40-49 can be observed starting in 2017. Otherwise, all other age groups showed a steady rate (Figure 2). 

**Figure 3:**
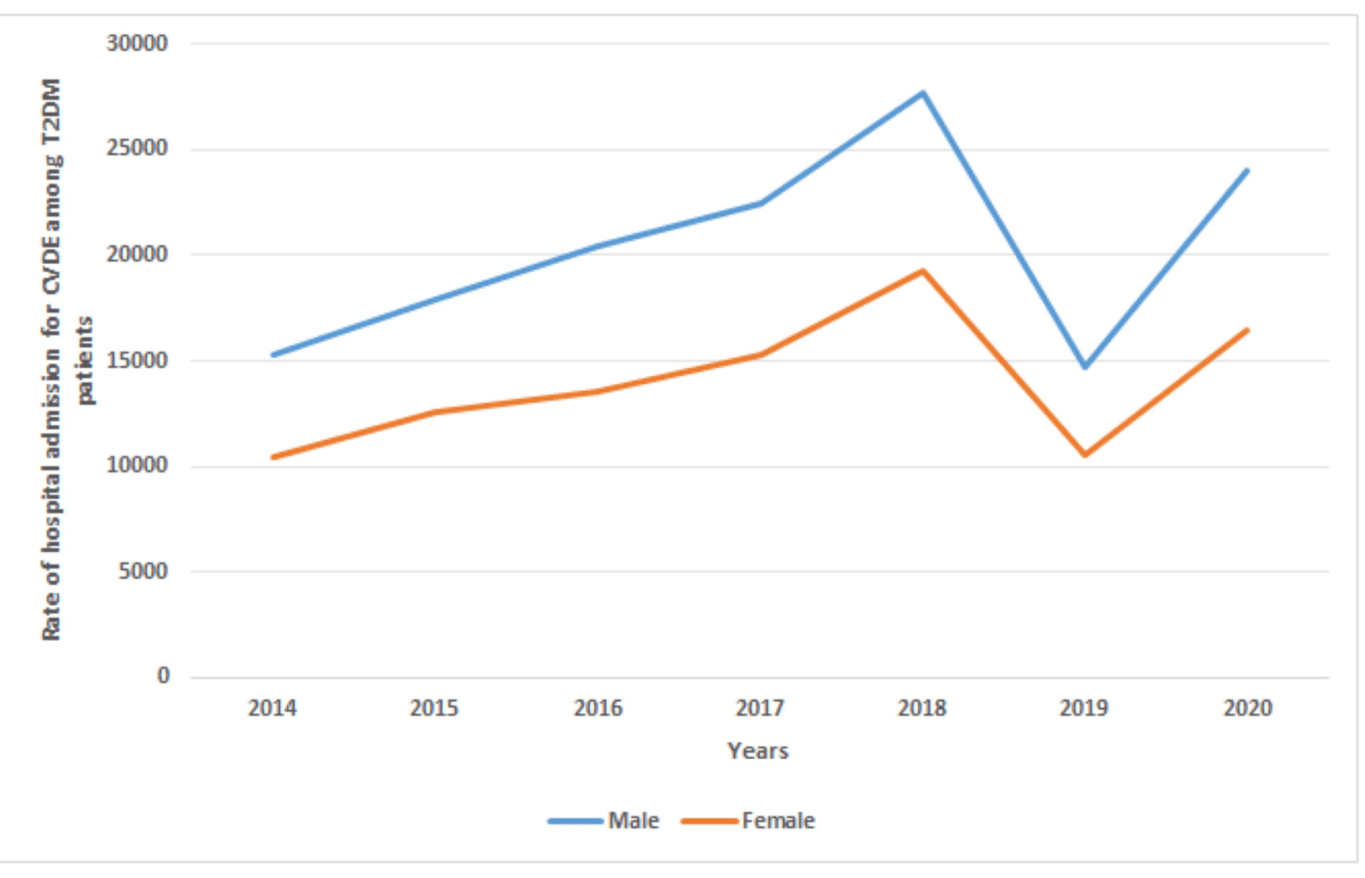
Rate of hospital admissions for all CVDs among T2DM patients in Malaysia, stratified by gender. CVD, cardiovascular disease; CVDE, cardiovascular disease event; T2DM, type 2 diabetes mellitus

A total of 682,948 CVD episodes were reported in public hospitals in Malaysia during the study period. Of the study patients, 35.1% had a previous history of or had been diagnosed with T2DM. In the T2DM population, males accounted for 59.3% of the total number of patients with CVD, representing 142,466 hospital admission episodes, with an average of 20,352 admissions per year. The CVD rate remained consistently high in males, with a mean of 59.1% (ranging from 58.1% to 60.1%) throughout the years (Figure 3).

Hospital admission rates for CVDEs by disease, gender, and age group

**Figure 4:**
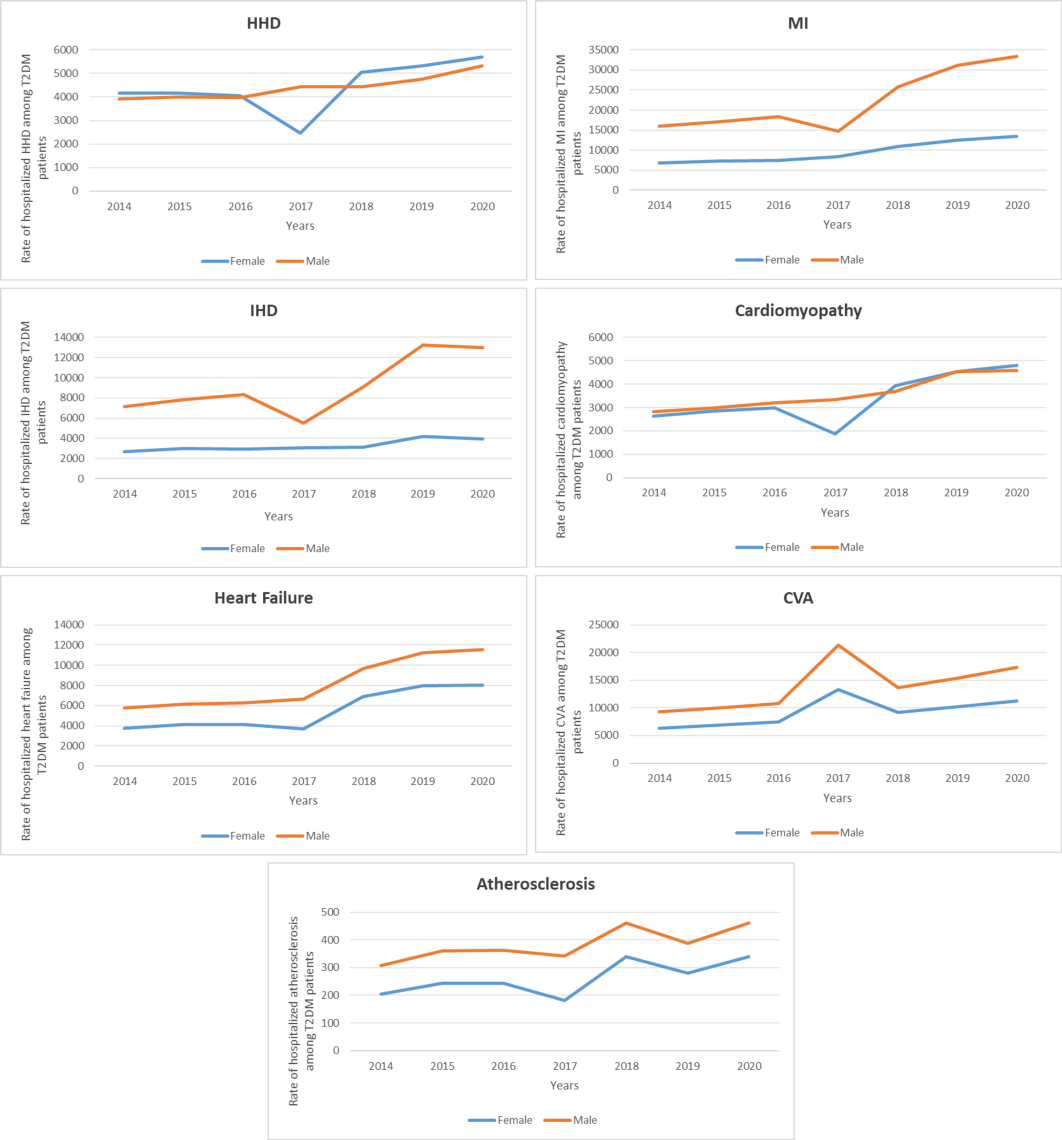
Hospital admission rates for CVD among T2DM patients in Malaysia, stratified by gender. CVD, cardiovascular disease; T2DM, type 2 diabetes mellitus; HHD, hypertensive heart disease; MI, myocardial infarction; IHD, ischemic heart disease; CVA, cerebrovascular accident

**Figure 5:**
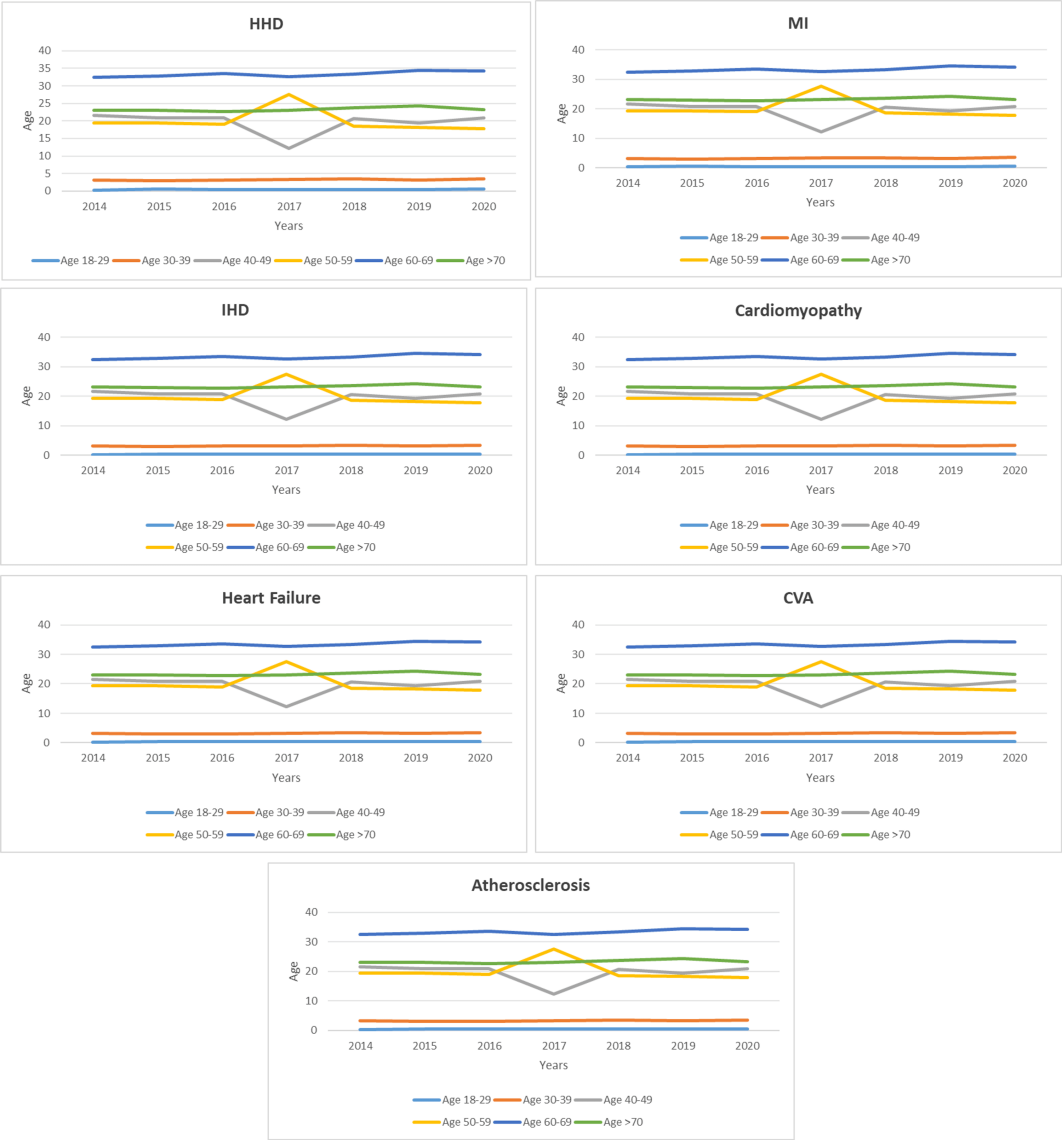
Hospital admissions rates for CVD among T2DM patients in Malaysia, stratified by age group. CVD, cardiovascular disease; T2DM, type 2 diabetes mellitus; HHD, hypertensive heart disease; MI, myocardial infarction; IHD, ischemic heart disease; HF, heart failure; CVA, cerebrovascular accident

The rates of CVDE were higher among males compared to females except for HHD and cardiomyopathy with conduct disorder, which were more common among females (Figure 4). However, the age group 60-69 is more dominant across all CVDEs (Figure 5).

Cost of CVDE among T2DM: 2014-2020

Table 2 shows the median and total cost for all CVDE among T2DM from the years 2014 to 2020. The overall inpatient expenditure for seven years duration was approximately RM 1,301,583,310.00 (median cost per case range RM 3,494.42 to RM 5,642.63). The trend of admissions is increasing every year (Table 2), with the median total hospital expenditure higher in IHD patients with T2DM, which increased by 55.5% from 2014 to 2020 (from RM 4,187.98 to RM 6,510.43), followed by MI, which increased by 8% (from RM 3,881.80 to RM 4,211.18). However, the costs for cardiomyopathy and HF showed decreasing trends of 24.4% and 17.6%, respectively (Table 3).

**Table 2:** Median cost per case for CVD among T2DM patients from 2014 to 2020, along with the total cost for each year (RM). RM, Ringgit Malaysia

Years	*N*	Median cost per case (RM)	Minimum cost (RM)	Maximum cost (RM)	Total cost for all cases (RM)
2014	25,715.00	4,992.93	2,138.69	23,770.80	130,713,274.56
2015	30,450.00	5,204.57	2,480.96	23,182.65	163,854,743.43
2016	34,001.00	5,642.63	2,629.72	60,220.75	286,985,256.77
2017	37,757.00	3,807.89	1,662.63	33,691.05	155,546,655.25
2018	46,947.00	3,494.42	2,578.67	35,657.86	202,038,571.37
2019	25,261.00	4,690.69	1,932.75	106,357.88	143,340,238.71
2020	40,480.00	4,913.96	2,545.59	75,654.16	219,660,161.87

**Table 3:** Median cost per case for each CVD among T2DM from 2014 to 2020 (RM). HHD, hypertensive heart disease; MI, myocardial infarction; IHD, ischemic heart disease; CVA, cerebrovascular disease; RM, Ringgit Malaysia

Years	HHD	MI	IHD	Cardiomyopathy	Heart failure	CVA	Atherosclerosis
2014	2,826.23	3,881.80	4,187.98	4,992.46	5,773.16	5,223.94	6,661.39
2015	3,523.40	4,033.46	4,033.46	4,603.67	5,464.22	5,767.75	7,420.24
2016	2,629.72	5,642.63	22,417.33	8,007.50	8,860.46	4,652.92	5,595.24
2017	3,595.74	3,595.74	3,807.89	3,807.89	3,807.89	3,922.87	3,807.89
2018	2,578.67	3,494.42	6,007.36	3,451.23	4,178.64	3,864.05	4,990.29
2019	2,849.29	4,521.58	6,928.41	4,598.89	4,690.68	5,452.63	5,708.21
2020	2,994.54	4,211.18	6,510.43	4,012.73	4,949.65	5,645.23	6,932.60

**Figure 6:**
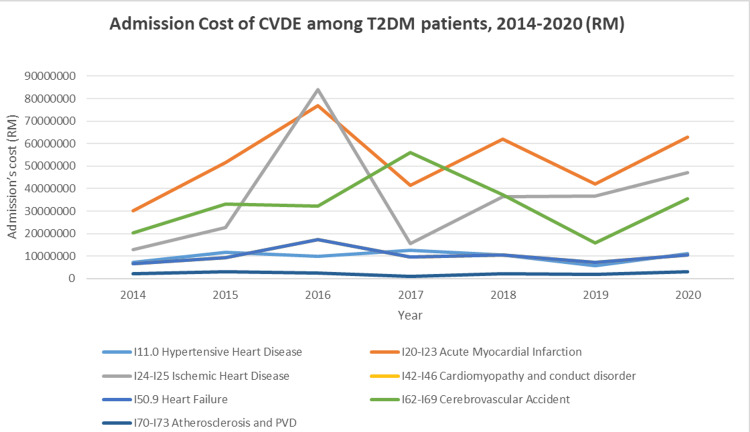
Total cost per cardiovascular disease among T2DM from 2014 to 2020 (RM). CVDE, cardiovascular disease event; T2DM, type 2 diabetes mellitus; PVD, peripheral vascular disease; RM, Ringgit Malaysia

The financial burden of CVD among T2DM patients showed considerable variability from 2014 to 2020. While the median cost per case fluctuated, the total cost was significantly influenced by the number of cases each year (Figure 6). The yearly trends were divided into 2014-2016, 2017-2018, and 2019-2020. From 2014 to 2016, the median cost per case increased from RM 4,992.93 in 2014 to RM 5,642.63 in 2016. The total cost also showed a significant rise, peaking at RM 286,985,256.77 in 2016. In 2017-2018, there was a noticeable decrease in the median cost per case, dropping to RM 3,807.89 in 2017 and further to RM 3,494.42 in 2018. However, the number of cases increased, leading to a relatively high total cost. Meanwhile, the median cost per case rose again in 2019-2020, reaching RM 4,913.96 in 2020. The total cost also increased, with a notable rise in the number of cases in 2020. The minimum cost per case showed some fluctuations but remained relatively stable compared to the maximum cost per case, which varied significantly, especially in 2019 and 2020. The maximum cost per case peaked in 2019 at RM 106,357.88, indicating a few extremely high-cost cases. The total cost for CVD among T2DM patients varied each year, with the highest total cost recorded in 2016 (RM 286,985,256.77) and the lowest in 2014 (RM 130,713,274.56). The number of cases each year played a crucial role in determining the total cost, with 2018 having the highest number of cases (46,947) and 2019 the lowest (25,261).

Factors determining the cost of CVDE among T2DM patients

The multiple logistic regression was used to determine the factors influencing the cost of treatment, utilizing the Malaysian DRG costing data. The logistic regression analyses reveal significant associations between demographic and clinical variables and the likelihood of CVDEs as stated in Table 4.

**Table 4:** Multiple logistic regression between independent variables as listed with treatment costs. CI, confidence interval; OR, odds ratio; LOS, length of stay; CVDE, cardiovascular disease event; HHD, hypertensive heart disease; MI, myocardial infarction; IHD, ischemic heart disease; HF, heart failure; CVA, cerebrovascular accident

Variable	Multiple logistic regression				*P*-value
	Coefficient	Adjusted OR	95% CI		
			Lower bound	Upper bound	
Age (Years)					
19-29	0.071	1.074	0.916	1.258	<0.001
30-39	0.086	1.089	1.027	1.155	0.004
40-49	0.215	1.24	1.204	1.278	<0.0001
50-59	0.055	1.057	1.026	1.088	<0.0001
60-69	0.082	1.086	1.058	1.114	<0.0001
>70	Reference				
Gender					
Male	0.122	1.13	1.107	1.152	<0.0001
Female	Reference				
Ethnicity					
Malay	Reference				
Chinese	0.126	1.135	1.103	1.167	<0.0001
Indian	Not significant				
Others	0.128	1.136	1.086	1.189	<0.0001
LOS					
<5 days	-0.263	0.769	0.752	0.785	<0.0001
>5 days	Reference				
Severity					
Mild	Reference				
Moderate	0.543	1.721	1.682	1.76	<0.0001
Severe	2.341	10.393	10.074	10.722	<0.0001
CVDE					
HHD	Reference				
MI	2.151	8.591	8.016	9.209	<0.0001
IHD	3.136	23.018	21.428	24.725	<0.0001
Cardiomyopathy	2.113	8.272	7.65	8.946	<0.0001
HF	2.351	10.494	9.781	11.258	<0.0001
CVA	2.162	8.688	8.096	9.323	<0.0001
Atherosclerosis	3.977	53.383	47.664	59.789	<0.0001

Patients aged 40-49 years had 24% higher odds of incurring elevated costs for CVD treatment compared to those over 70, while individuals aged 60-69 and 50-59 had 8.6% and 5.7% higher odds, respectively. Males were 1.13 times more likely to incur high treatment costs than females. Compared to Malays, individuals from Chinese and other ethnic groups exhibited 13% higher odds of experiencing increased treatment costs. Patients with a hospital stay of less than five days were 23.1% less likely to incur high treatment costs than those with longer stays. Additionally, patients classified with severity level 3 (severe) were 10 times more likely to face higher treatment costs than those with severity level 1 (mild). Finally, patients admitted for atherosclerosis, with peripheral vascular disease (PVD) and MI, were 53 and 21 times more likely, respectively, to incur higher CVD costs compared to those admitted for HHD.

## Discussion

The admission trends for CVD among patients with T2DM in Malaysia are driven by the increasing prevalence of T2DM, which has significantly contributed to the rising burden of NCDs in the population [5]. Over the years, there has been a steady increase in comorbidities such as hypertension and dyslipidemia among T2DM patients. Additionally, the incidence of cardiovascular complications, including IHD, nephropathy, and retinopathy, has escalated significantly within this population. The analysis of the median per-patient cost (PPC) for CVDEs in T2DM patients from 2014 to 2020 reveals notable trends. As shown in the chart, the costs associated with IHD and HF are consistently high across the years, with a significant spike in IHD-related costs in 2016, reaching nearly RM 25,000. Costs for other conditions, such as cardiomyopathy and CVAs, have also shown a steady increase, while costs related to HHD and atherosclerosis with peripheral vascular disease (PVD) remain substantial but less pronounced.

This might have been due to increased stress levels, as modern lifestyles, work pressures, and urbanization led to higher stress, which is closely associated with cardiovascular risks. Chronic stress contributes to elevated blood pressure and inflammation, both of which exacerbate the risk of IHD and HF. Financial burden and delayed diagnosis coupled with suboptimal management can accelerate the disease progression. The rising cost of living and healthcare expenses may prevent some individuals from accessing timely medical care or adhering to treatment regimens for diabetes and its complications, leading to worsening conditions over time. A sedentary lifestyle and poor dietary habits like increasing adoption of sedentary lifestyles and unhealthy diets high in sugar and fat contribute to obesity, dyslipidemia, and hypertension, compounding the risk for CVDs. With a growing aging population in Malaysia, the risk of both IHD and HF naturally increases, as age is a significant risk factor for CVDs. Furthermore, improved diagnostic capabilities, advances in diagnostic tools, and increased awareness may lead to more cases being identified, contributing to the rise in reported IHD and HF cases.

Factors that are significantly associated with the costs of CVD admissions among T2DM patients in Malaysia encompass hospital type, diabetic status, gender, ethnicity, severity level, type of CVD, patient age, and length of stay (Table 4). Overall, the increasing burden of cardiovascular risk factors such as diabetes, hypertension, and dyslipidemia, combined with inadequate knowledge and prevention practices, are primary drivers of the rising hospitalization rates for CVD among T2DM patients in Malaysia.

In Malaysia, the costs associated with CVDs in patients with T2DM are influenced by various factors. Significant contributors to admission costs include hospital type, diabetic status, gender, ethnicity, severity level, type of CVD, patient age, and length of stay [12]. The estimated annual cost of diabetes in Malaysia is approximately USD 600 million, with age, hospital type, length of inpatient stay, and frequency of outpatient visits having a significant impact on these costs [13]. Predictors of CVD in T2DM patients include increased age, lower HDL-C levels, and low to moderate levels of physical activity [14]. In Selangor, the cost of treating diabetes mellitus in 2004 was estimated at RM 18,956,021.51, which accounted for 3.3% of total state health expenditure, with 60.2% allocated to outpatient care and 39.8% to inpatient care [15]. Patient follow-up costs were estimated at RM 459 per year, while complication costs ranged from RM 479 for retinopathy to RM 42,362 for nephropathy [16].

Research on gender and ethnicity as factors influencing hospital admission costs reveals significant disparities. Depression in HF patients increases hospital costs by 22% across all gender and ethnic groups [17]. Race, income, and age are significant determinants of hospital charges, while gender is not [18]. However, males have higher odds of receiving various procedures compared to females, and White patients generally have higher odds than minorities for many procedures [19]. In a low-income diabetic cohort, hospitalization rates increased with age and the interaction of male gender and age. Non-Hispanic White patients had higher rates than African American patients, while other ethnicities had lower rates [20]. These studies highlight the complex interplay of gender, ethnicity, and other factors in hospital admission costs and procedure utilization, emphasizing the need for further research and targeted interventions to address disparities.

By analyzing data from a tertiary care hospital, we have, for the first time in Malaysia, quantified the costs associated with public hospital care for individuals with CVD and T2DM, along with their primary drivers. The examination of hospital admission data and costs related to CVD in diabetes patients provides critical insights. The total costs for the analyzed period were substantial, exceeding RM 1.3 billion, with the median cost per hospitalization episode for CVD among patients with diabetes ranging from RM 3,494.42 to RM 5,642.63, irrespective of age, complications, or type of CVD.

Diabetes prevalence and costs are rising globally, with significant impacts on healthcare systems. Annual diabetes treatment costs vary widely, ranging from $15 to over $500 for medications alone, with insulin prices differing substantially [21]. CVD-related costs per episode typically range from $500 to $1,500, with costs for stroke and coronary heart disease potentially exceeding $5,000 [22]. In Europe, diabetes prevalence was estimated at 8.5% in 2013, affecting 56 million people, with projections of 66 million by 2035 [23]. The global prevalence in 2021 was 10.5%, expected to reach 12.2% by 2045 [24]. In Romania, diabetes management costs increased between 2000 and 2017, with the average annual cost per patient reaching 215 EUR [25]. A study of eight European countries estimated the total direct medical costs of T2DM at 29 billion EUR annually, with hospitalizations accounting for 55% of costs [26]. Global diabetes-related health expenditures were estimated at 966 billion USD in 2021, projected to reach 1,054 billion USD by 2045 [24]. These findings highlight the growing economic burden of diabetes on healthcare systems worldwide.

Despite the substantial economic burden, evidence regarding treatment costs in low- and middle-income countries (LMICs) remains limited and of low quality, with most studies focusing on middle-income countries [27]. To mitigate this burden, experts recommend implementing cost-effective interventions that prioritize affordability and feasibility. The World Health Organization suggests a core set of interventions costing $1 to $3 per capita annually, depending on the country’s income level [28].

Research indicates that diabetic patients face a significantly higher risk of cardiovascular complications compared to non-diabetic individuals [29,30]. These complications contribute to increased healthcare utilization and associated costs [31,32]. Diabetic patients who experience cardiovascular events incur greater direct medical costs and longer hospital stays than their non-diabetic counterparts [33]. The incidence of CVD is notably higher in individuals with T2DM than in non-diabetic individuals, leading to prolonged hospitalizations and increased costs for specific complications [34,35]. Among T2DM-related complications, macrovascular diseases represent the highest management costs, with MI incurring the highest cost in the event year (USD 4,528.37) and HF in subsequent years (USD 524.79) [36]. Additionally, the economic burden is particularly pronounced for patients experiencing stroke complications [37].

However, when comparing diabetes alone to ASCVD, diabetes is associated with lower healthcare expenditures and resource utilization [38]. The combination of ASCVD and diabetes results in the highest healthcare costs, underscoring the necessity for prevention and early management. Notably, when controlling for various factors, total admission costs for diabetic and non-diabetic patients are comparable, although diabetic patients with congestive HF tend to experience longer hospital stays [39]. These findings elucidate the complex interplay between diabetes, CVD, and healthcare costs. Our results complement the recently published data on the evolution of costs of CVDE management among diabetes inpatient settings in Malaysia, which showed an increasing trend between 2014 and 2020. As compared to other countries, the average costs per episode of hospitalization were significantly lower in our country.

Diabetes management programs may reduce hospital utilization and costs for diabetes-related complications in the short term, but their impact on CVD costs remains uncertain [40,41]. Nonpharmacological approaches, such as exercise and diet, can help reduce medication costs for diabetic patients with cardiovascular complications [34]. The research highlights the need to examine data related to patient admissions to enhance the management of diabetes and lower healthcare expenses. Essentially, by understanding patterns and factors in admission data, healthcare providers can develop better strategies for treating diabetes, which could lead to improved patient outcomes and reduced costs for the healthcare system.

Although we attempted to provide a detailed analysis of inpatient medical costs, our analysis has limitations. First, only the costs of persons discharged with a diagnosis of diabetes were available for this analysis. If the admission is not related to diabetes or the diabetic ICD-10 code is not recorded, we cannot capture that person. Another limitation is that we did not have the costs split per medication and investigation. The lack of granular data on socioeconomic or behavioral factors is one of the limitations. No data on glycemic control and other sociodemographic variables of participants were included in the database provided. For example, previous studies showed that hospitalization costs were influenced by smoking habits, with 24% to 35% higher costs for current and former smokers as compared to never-smokers [42,43]. The availability of information on these risk factors may have provided deeper insights into the figures found. The public-center design is another limitation of the study. Although our hospital functions as both a secondary and tertiary care hospital, the costs depicted may overestimate the costs associated with diabetes care in other private hospitals.

## Conclusions

This study highlights critical trends in hospital admissions and the escalating costs associated with CVD among patients with T2DM. The findings underscore a troubling rise in both the prevalence of cardiovascular complications and the resulting financial strain on the healthcare system. These trends are driven by factors such as the increasing prevalence of T2DM, the presence of comorbid conditions like hypertension and dyslipidemia, and demographic factors such as aging and urbanization.

Understanding these patterns is vital for policymakers and healthcare providers to design and implement targeted interventions aimed at reducing the impact of CVD on this high-risk population. Policymakers should prioritize expanding access to affordable screening and diagnostic services for early detection of cardiovascular risks, especially in rural and underserved areas. Programs such as Skim Perubatan Madani and PeKa B40 should be promoted widely. Additionally, investing in public awareness campaigns about the risks of unmanaged diabetes and the benefits of preventive measures could significantly impact outcomes.

Prevention strategies specific to Malaysia should include promoting culturally appropriate dietary modifications, such as reducing the consumption of high-sugar foods and processed carbohydrates, which are common in the local diet. Initiatives to encourage physical activity, such as community-based fitness programs or subsidized memberships at local recreational centers, could help address sedentary lifestyles. Addressing financial barriers to healthcare by providing subsidies for diabetes-related medications and treatments can also improve patient adherence to treatment plans and prevent complications.

Moreover, implementing workplace wellness programs and stress management workshops could help mitigate lifestyle-related risk factors. Collaborating with local organizations, including nongovernmental organizations and community leaders, can amplify outreach efforts and ensure that interventions are culturally sensitive and accessible.

Finally, the study underscores the necessity for further research into cost-effective, scalable healthcare solutions tailored to Malaysia’s unique socioeconomic and cultural landscape. Proactive steps in prevention and management not only have the potential to improve patient outcomes but also to reduce the financial burden on the healthcare system, ensuring its sustainability in the face of rising demand.
